# Exploration of transitional life events in individuals with Friedreich ataxia: Implications for genetic counseling

**DOI:** 10.1186/1744-9081-6-65

**Published:** 2010-10-27

**Authors:** V Brook White, Jennifer R Leib, Jennifer M Farmer, Barbara B Biesecker

**Affiliations:** 1 Clinical Genetics, Carolinas Medical Center, PO Box 32861, Charlotte, NC 28232-2861, USA; 2 HealthFutures, Washington, DC, USA; 3 Friedreich's Ataxia Research Alliance, Exton, PA, USA; 4 National Human Genome Research Institute, National Institutes of Health, Bethesda, MD, USA

## Abstract

**Abstract:**

**Background**

Human development is a process of change, adaptation and growth. Throughout this process, transitional events mark important points in time when one's life course is significantly altered. This study captures transitional life events brought about or altered by Friedreich ataxia, a progressive chronic illness leading to disability, and the impact of these events on an affected individual's life course.

**Methods**

Forty-two adults with Friedreich ataxia (18-65y) were interviewed regarding their perceptions of transitional life events. Data from the interviews were coded and analyzed thematically using an iterative process.

**Results**

Identified transitions were either a direct outcome of Friedreich ataxia, or a developmental event altered by having the condition. Specifically, an awareness of symptoms, fear of falling and changes in mobility status were the most salient themes from the experience of living with Friedreich ataxia. Developmental events primarily influenced by the condition were one's relationships and life's work.

**Conclusions**

Friedreich ataxia increased the complexity and magnitude of transitional events for study participants. Transitional events commonly represented significant loss and presented challenges to self-esteem and identity. Findings from this study help alert professionals of potentially challenging times in patients' lives, which are influenced by chronic illness or disability. Implications for developmental counseling approaches are suggested for genetic counseling.

**Background:**

Human development can be described in terms of key transitional events, or significant times of change. Transitional events initiate shifts in the meaning or direction of life and require the individual to develop skills or utilize coping strategies to adapt to a novel situation [1,2]. A successful transition has been defined as the development of a sense of mastery over the changed event [3].

Transitions can be influenced by a variety of factors including one's stage of development, such as graduation from high school, historical events, including war, and idiosyncratic factors, such as health status [4,5]. Of particular interest in the present study are transitional life events, brought about or altered by progressive chronic illness and disability, and the impact of these events on the lives of affected individuals.

It has been recognized that the clinical characteristics of a chronic illness or disability may alter the course and timing of many developmentally-related transitional events [6]. For example, conditions associated with a shortened lifespan may cause an individual to pursue a career with a shorter course of training [6]. Specific medical manifestations may also promote a lifestyle incongruent with developmental needs [6,7]. For example, an adolescent with a disability may have difficulty achieving autonomy because of his/her physical dependence on others.

In addition to the aforementioned effects of chronic illness and disability on developmentally-related transitional events, a growing body of literature has described disease-related transitional events: those changes that are a direct result of chronic illness and disability. Diagnosis has received attention as being a key disease-related transitional event [8,9]. Studies have also noted other disease transitions related to illness trajectory [10], as the clinical features of the disease may require the individual to make specific adaptations. Disease-related events have also been described in terms of accompanying psychological processes, such as one's awareness of differences brought about by illness [11].

While disease-related events are seemingly significant, the patient's perception of the events is varied. Some events may be perceived as positive experiences for the individual. For example, a diagnosis may end years of uncertainty. Some individuals may perceive these transitional events as insignificant, as they have accommodated to the continual change brought about by a chronic disease [12,13].

The aforementioned impact of disability and chronic illness on transitional events may create psychological stress. Developed by Lazarus and Folkman, the Transitional Model of Stress and Coping describes the process of adaptation to a health condition [14]. This model purports that individuals first appraise a stressor and then utilize a variety of coping strategies in order to meet the stressor's demands [14]. Thus, in the context of chronic illness, the ability of the individual to cope successfully with the stress of a health threat contributes to the process of overall adaptation to the condition.

The process of adaptation can be more complex when the chronic illness or disability is progressive. Each transition brought about or altered by the disability may also represent additional loss, including the loss of future plans, freedom in social life and the ability to participate in hobbies [15]. These losses may be accompanied by grief, uncertainty, and a continual need for adaptation [16,17].

Friedreich ataxia (FRDA) is one example of a progressive disorder, leading to adolescent and adult onset disability. To better understand patients' perceptions of key transitional events and the factors perceived to facilitate progression through these events, individuals with FRDA were interviewed.

FRDA is a rare, progressive, neurodegenerative disorder affecting approximately one in 30,000 people in the United States [18]. It equally affects both men and women. Individuals with FRDA experience progressive muscle weakness and loss of coordination in the arms and legs. For most patients, ataxia leads to motor incapacitation and full-time use of a wheelchair, commonly by the late teens or early twenties. Other complications such as vision and hearing impairment, dysarthria, scoliosis, diabetes mellitus and hypertrophic cardiomyopathy may occur [19,20]. Cardiomyopathy and respiratory difficulties often lead to premature death at an average age of 37 years [21]. Currently, there are no treatments or cures for FRDA. Little is known about the specific psychological or psychosocial effects of the condition.

FRDA is an autosomal recessive condition. The typical molecular basis of Friedreich ataxia is the expansion of a GAA trinucleotide repeat in both copies of the *FXN *gene [22]. Age of onset usually occurs in late childhood or early adolescence. However, the availability of genetic testing has identified affected individuals with an adult form of the condition. This late-onset form is thought to represent approximately 10-15% of the total FRDA population [23].

Health care providers of individuals with progressive, neurodegenerative disorders can help facilitate their patients' progression through transitional events. Data suggest that improvements should be made in the care of these individuals. Shaw et al. [24] found that individualized care that helps to prepare patients for transition is beneficial. Beisecker et al. [25] found that patients desire not only physical care from their providers, but also emotional and psychosocial support.

Genetic counselors have an important opportunity to help patients with neuromuscular disorders progress through transitional events, as several of these conditions have a genetic etiology. Genetic counselors in pediatric and adult settings often develop long-term relationships with patients, due to follow-up care. This extended relationship is becoming increasingly common as genetic counselors move into various medical sub-specialties, such as neurology, ophthalmology, oncology and cardiology.

The role of the genetic counselor in addressing the psychosocial needs of patients has been advocated, but rarely framed in the context of developmental events [26]. Data suggest that patients may not expect a genetic counselor to address psychosocial needs [27]. In a survey of genetic counseling patients, Wertz [28] found a majority of respondents understood genetic conditions to have a moderate to serious effect on family life and finances, while almost half perceived there to be an effect on the spouse, quality of life, and the relationship between home and work. However, these topics were reportedly not discussed within genetic counseling sessions [27,28]. Overall, there is limited information about the experiences of transitional life events in FRDA, as well as a lack of recommendations for genetic counselors and other health care providers to assist patients through these events.

Our study investigated perceptions of patients with Friedreich ataxia to 1) identify key transitional events and specific needs associated with events; 2) describe perception of factors to facilitate progression through the identified events; and 3) explore the actual or potential role of the health care provider in facilitating adaptation to the identified events. Data were used to make suggestions for developmental genetic counseling approaches in the context of ongoing care of clients with hereditary, progressive, neurodegenerative conditions.

## Methods

### Participants

Individuals, eighteen years of age and older, carrying the diagnosis of Friedreich ataxia, were eligible for participation in this study.

The study was approved by the National Human Genome Research Institute (NHGRI) Institutional Review Board.

### Recruitment

Recruitment occurred at the National Ataxia Foundation's (NAF) conference and the National Institutes of Neurological Disorders and Stroke (NINDS), Neurogenetics Branch. An NINDS staff member sent letters to individuals who had previously requested information regarding research studies in FRDA. Individuals interested in participating contacted one of the researchers and written informed consent was obtained prior to participation.

### Data collection

A semi-structured interview guide consisted of open-ended questions, as well as specific probes designed to elicit information about the participant's key transitional life events, his/her sources of support, and experience with health care providers. Specifically, participants were asked to identify key transitions, and to elaborate on the details of the experience. Demographic information, including gender, age, education and marital status was collected. FRDA related information, such as age of diagnosis and mobility status, was also obtained to provide a fuller description of the disease context for each participant. Interviews averaged 53 minutes (from 37 to 72 minutes). In-person interviews were conducted for 19 individuals present at the NAF conference. Twenty-three individuals recruited from NINDS participated in a telephone interview. All interviews were tape-recorded and transcribed verbatim. Transcripts were checked for accuracy by the interviewer.

### Data analysis

Data from the interviews were analyzed thematically. Using a template analysis style [29] an initial list of codes, based on the present literature regarding transitional events and chronic illness, was constructed prior to conducting interviews. New codes were then added and revised as data were obtained. Codes were then applied to all interviews to provide both context and variation for meaningful themes. Software for analyzing qualitative data, QSR-NUDIST [30], was used to facilitate the coding process. One-third of the transcripts were read and coded by a second coder (BBB) for inter-coder reliability which was high (> 0.9). Themes were summarized across transcripts on an interpretive level.

## Results

### Sample characteristics

Forty-two individuals participated in the study. Recruitment stopped when data reached saturation, that is, data redundancy was achieved and little new information was obtained. Table 1 describes the sociodemographics of the participants. Overall, the participants were single, white, non-Hispanic, with some college education. The majority of the sample population used a wheelchair and nearly half of the participants had a sibling with FRDA.

**Table 1:** Characteristics of the research sample

Gender	N (___%)
Male	19 (45%)
Female	23 (55%)
**Age**	
Average (range)	38.4y (18-65y)
**Race**	
White, non Hispanic	41 (98%)
Hispanic	1 (2%)
**Current marital status**	
Single, never married	22 (52%)
Married	13 (31%)
Divorced	7 (17%)
**Number of children**	
None	21 (50%)
One - two	19 (45%)
Three or more	2 (5%)
**Highest education level completed**	
High school	3 (7%)
Some college	21 (50%)
College degree	11 (26%)
Graduate degree	7 (17%)
**Age of onset of symptoms**	
Average (range)	19.8y (5-47y)
**Age of diagnosis**	
Average	28.9y (9-62y)
**Assistive devices**	
Participants using a wheelchair	25 (60%)
Average length of time using a wheelchair	6.3y
Average age of obtaining a wheelchair	28.3y

### Transitional life events

All 42 participants identified transitional events and described the influence of Friedreich ataxia on those times of change. Transitional events were either a direct outcome of having FRDA, or a developmentally-related event altered by the presence of the condition. A series of transitions including recognition of symptoms, fear of falling and changes in mobility status, embodied the Friedreich's experience. These transitions directly influenced one's life work and relationships. Overall, identified events had a significant impact on self-image, life activities, and future planning. Identified times of change led to the assessment of a novel situation, and implementation of coping strategies toward adaptation or re-adaptation. Transitional events are summarized in Appendix 1.

#### The Friedreich experience

The 'Friedreich experience' was marked by a series of transitions corresponding to clinical progression. The series began with a self-awareness of symptoms and a subsequent fear of falling. Eventually, decisions regarding assistive devices were necessary. Each component of the Friedreich experience was marked by loss, with the exception of transitioning to a wheelchair, which was commonly associated a new found discovery of freedom and independence.

##### Recognition of symptoms

A personal awareness of symptoms marked the initial life change brought about by FRDA. Interestingly, this change did not correspond with receiving a diagnosis. Rather, participants placed emphasis on the period of time when the diagnosis was unknown, but symptoms were apparent. Many individuals encountered self-doubt as they noted physical changes. When doctors could not identify the medical etiology, support persons ceased to aid in the pursuit of a diagnosis, and participants experienced feelings of isolation. According to one female participant,

I was walking around thinking, it's in your head, nobody else can see it, but I'm feeling this way...I went I would say about a year and a half thinking that I was crazy. When I did get the diagnosis it was by myself, because my father didn't want to go to the doctor with me. Nobody thought that there was anything wrong. (female, age 32 years)

The recognition of symptoms led individuals to become increasingly self-conscious, as others commonly perceived them to be clumsy or drunk. Many recalled being teased, while others noted the struggle to perform certain tasks. One participant reported,

When I knew something was wrong, nobody else believed me, my parents didn't, my doctor -- they told me I was clumsy and that I'll grow out of it. (female, age 22 years)

##### Fear of falling

The next temporal transition within the experience of living with FRDA salient to participants was the fear of falling. For many, this concern arose from a previous fall, while others reported times they had nearly fallen. As one participant explained,

I probably fall once a year. I have gotten away with only tearing my ankle. But, in the back of my mind I always knew I was going -- there was going to be another incidence. One of these times, my luck is going to run out. (male, age 42 years)

The fear of falling greatly affected one's willingness to participate in activities. Many reported not participating in the same activity performed during a previous accident. As one male participant noted,

I've done things for as long as I could until I had an incident where I had fell and injured myself where I knew I couldn't do them, you know, anymore...it wasn't impossible but the risk of injury was getting too great. (male, age 42 years)

Others expressed hesitation in going to crowded public places, including shopping malls, sporting events and restaurants. The embarrassment, anxiety and concern about social stigma corresponded with an inability to enjoy a visit to these venues.

You're more concerned about others watching you falling in front of someone and embarrassing yourself. (male, age 34 years)

##### Mobility status

Changes involving mobility status represent the final transition within the Friedreich experience. These changes were associated with the use of assistive devices, including canes, walkers, and wheelchairs.

The initial challenges associated with using an assistive device were perceived stigma, and potential decline in freedom and independence. Stigma was related to individuals' associations of an assistive device with looking old, giving up the fight, or the loss of intellectual capacity. According to these participants,

It's embarrassing to go out in public with a walker, being I'm only 23 years old. (male, age 23 years)

I got treated really funny by like people that think because you're in a wheelchair you lose your brain power. (female, age 46 years)

Additionally, many were concerned that it would be difficult to make a positive first impression, particularly when going for a job interview or meeting new people. As one participant stated,

And even if I have a degree in something and I'm in a wheelchair do you know how challenging that would be for someone to hire me? (female, age 32 years)

Notably, after an initial adjustment period many participants remarked on positive aspects of an assistive device. Individuals transitioning to a cane or walker noted that using the device reduced stigma. Several participants echoed the words of this individual,

I had a cane. The only reason I carried it was so that people wouldn't say I was drunk. (male, age 29 years)

Likewise, after an initial adjustment period, individuals using a wheelchair noted that it provided increased independence and freedom.

People always say -- make it seem like you -- almost like you're giving up. And to me, after I tried it, it was like gaining -- because I got to do things that I couldn't do. (female, age 46 years)

You know people say confined to a wheelchair. But I think if I'm in a chair without wheels then I'm really confined. (female, age 33 years)

Freedom and independence was increased, as moving from place to place was not as mentally or physically exhausting as it was prior to using a wheelchair. Many participants also felt a greater sense of safety. These factors led to the ability to go places they previously avoided. For example,

My personality seemed to come out more and -- I just felt like I could be myself and talk... I was losing some mobility, but I was gaining lots. (male, age 24 years)

#### Developmental events

Participants also identified developmentally-related transitional events. Changes in one's work and primary relationships were the most salient events. Like the Friedreich-related transitions, change surrounding career and relationships initiated the assessment, coping and eventual adjustment to new challenges in life.

##### Relationships

Participants articulated several means by which relationships were influenced by FRDA. The primary relationships affected were dating, marriage and parenting.

Dating relationships were a common theme for the present study population, as greater than half of the individuals were single at the time of the interview (Table 1). Within these relationships, participants expressed difficulty finding dates because of the (1) physical presentation of FRDA, including balance and speech difficulties, (2) aspects of life influenced by FRDA, such as career and activities, (3) and the use of assistive devices. These three difficulties are illustrated in the following.

Being different can be a scarlet letter almost in the fact that -- to the girls, they wouldn't touch me with a 10-foot pole, you know. Because I wasn't typical...I don't blame the females for thinking the way they did or still do. You know, if they're wanting a man that can work and drive and all that, then that's their privilege. (male, age 32 years)

I think the hardest words that I heard was that [he couldn't] see a wheelchair in [his] future. (female, age 32 years)

Other individuals were involved in dating relationships, but found it challenging to explain to their partner the specifics of Friedreich ataxia. Conversations about the progression of the condition were particularly difficult. In the words of one participant,

They think you're in a chair because you were in an accident. And then you've got to share more information and they're like, "is it going to get worse?" (female, age 32 years)

Finally, participants spoke of previous dating relationships that had ended due to some aspect of FRDA. These events represented loss both emotionally and physically, as many reported using their date for balance and support. One's own acceptance of having FRDA was often complicated by a break-up, as it was perceived that the relationship ended because of the partner's difficulties in accepting the condition.

And I almost think that it's harder for somebody in a relationship to get involved. Because with me I have to accept it. I have no choice. And I think that for a potential spouse to get involved with that, there's concerns because they have a choice whether they want it in their life or not. And I think that's harder for the person. (female, age 32 years)

Like dating relationships, married participants reported that their relationships with their spouses were affected by FRDA. In many situations, a spouse was the primary support person. However, participants reported that it was difficult to communicate with one's spouse about issues generated by FRDA. Difficulty discussing the condition and its implications for the relationship led to feelings of isolation. As one participant explained,

He (husband) would always listen to me, but he doesn't share any of himself with me. (female, age 52 years)

There were also discrepancies within the couple surrounding physical expectations and abilities. For example, spouses sometimes wished the individual with FRDA could participate in a particular activity. These expectations often led to feelings of inadequacy and frustration.

I remember one time we were at a restaurant -- this waitress walked by with high heels and he (husband) goes, oh, I wish that one day you'd be able to wear heels like that. And I was like, well, that's not going to happen...and that's like, to me, hurtful. (female, age 50 years)

On the other hand, some spouses took on a protective role, not wanting their partner to participate in potentially dangerous activities. As one woman remarked,

It's justified that he (husband) would have fears. But, you know, I'm my own person. I'm independent. He doesn't have to worry so much. (female, age 26 years)

The parenting relationship was also influenced by Friedreich ataxia. Some individuals noted fear when assessing their ability to care for children. Others, more commonly, reported the inability to play or participate in activities with their children. For example,

This (FRDA) didn't really affect me until he got to be about four or five, and I could no longer play in the snow with him, or throw a baseball and have him hit the ball. (female, age 50 years)

These challenges led to both frustration and sadness, particularly when other parents took their children to activities that they were unable to participate in because of physical difficulties.

I feel the frustration...they have to go with the neighbor's kid's father to go hunting. So the -- the positive side is -- up to a point, we can share these passions, but then at a certain point, the frustration sets in again for me. (male, age 49 years)

Despite the frustrations experienced from not participating in their children's activities, most individuals noted that the parenting relationship was a primary motivation in their lives. Because their children 'needed them', they continued to face and overcome adversity. A common viewpoint is reflected in these quotes:

If I didn't have a daughter, I'm the first one to admit I probably wouldn't be here. I would have wrapped myself around a tree a long time ago. (male, 31 years)

I think if I wouldn't have had someone that depended on me... I think I would have given up trying to keep fighting, to stay around or to stay active as much as I was, you know. He needed me, no matter if I was a nurse, or had ataxia, or whatever the problem. It helped that I had him: that I knew I had to manage being his mother. (female, age 50 years)

Finally, some individuals had chosen not to have children because of FRDA. The decision not to have children was typically based on concern about progression of the condition and how it would affect their ability to parent the child, or about passing this recessive condition on to a child. Both scenarios were accompanied by feelings of loss and sadness, as illustrated in the following quote:

As bad as I would like to have a child, I could not fathom passing these genes down that will probably hurt my future generations. (male, 34 years)

Having another child it was like well, you know, I don't know how I'm going to be able to do things with the first two. I don't want to have another one and then not be able to do anything with that one. (female, age 33 years)

##### Life's work

The remaining category of transitional events revolves around one's life work. Primarily, these events encompass several aspects of career and education. Many participants noted that the careers they chose were influenced by FRDA. Decision-making regarding career choice was often a struggle between the desired career and the career that was perceived as physically possible both in the present and future. As one participant stated,

What your heart likes and what Friedreich's dictates are two different things. (male, age 32 years)

Individuals with an established career experienced changes in their job performance. Many individuals found ways to compensate so that co-workers or bosses would not notice the change. For example,

...I really couldn't keep up and I would stay and I would run down and clock out, and come back and finish my work. (female, age 50 years)

It was difficult for participants to balance their career and personal life. As the individual was working harder to compensate for difficulties related to the condition, he/she was also experiencing fatigue and weakness associated with FRDA. As one participant stated,

I'd given it the best nine hours of my day. The best part of me was given to my job. Because I physically couldn't - you know, if I worked all day, I mean, I would just come home and that was it for the night. (female, age 42 years)

Finally, many individuals reported that progression of the condition either led to quitting one's chosen line of work and changing to another position, or quitting work entirely, and taking disability or early retirement. This transition represented a substantial loss to the individual and was most often marked by feelings of guilt, inadequacy, sadness, frustration and the questioning of self identity.

But I did it (took another job). You know, when I think back now, when I think of how low I felt and how bad I felt not being a nurse. (female, age 50 years)

Additionally, these feelings were often compounded by the increased financial burden of not working, or the difficulties and time commitment when applying for disability.

#### Coping

Participants articulated a variety of factors that helped to facilitate their progression through the transitional events. Many of the efforts were adaptive coping strategies, such as focusing on the present and seeking out social support. Overall, a few participants reported maladaptive coping strategies, including the use of drugs and alcohol. Many individuals described their initial reaction to FA as denial, which reportedly diminished over time.

Participants reported cognitive strategies, such as maintaining focus on the present rather than the future. Utilizing this strategy shifted attention away from the condition and its prognosis onto present challenges.

Just take it one day at a time. Don't try to look down the road too far. Just keep your goals and your thoughts more present. (female, age 48 years)

Other common strategies involved compensating for physical difficulties. Participants reported trying to "look normal" by concentrating on performing a given task, placing their clinical features within a context, such as pretending to be drunk, or using their friends to maintain physical balance.

I spend a lot of energy at work trying to be as normal as possible. You know, I had one confidant who was very good at, -- if we were walking down the street or something and we had to go down a ramp, he would automatically go in front of me and I would sort of hold onto his coat. (female, age 46 years)

Individuals also used various forms of social support to help them through their times of change. Spouses, friends, and family were commonly reported to provide needed encouragement. Some found it helpful to become involved in support groups. Finally, several individuals reported receiving social and spiritual support through their faith and religious practices.

#### Health care providers

Participants reported a variety of positive and negative interactions with their health care providers (HCPs). Many negative experiences were associated with perceptions that their providers had limited knowledge of FRDA. Some participants felt that this limited knowledge had important implications for their health, while others were frustrated to spend time educating HCPs. These concerns are highlighted in the following:

When it came about finding out that it was Friedreich's, most of my doctors didn't know what it was and so 90% of my information I had to look up myself. (male, age 27 years)

Many participants also reported positive experiences with their HCPs. Positive experiences were often based on the provider's attributes, including their willingness to listen, compassion, and perceived interest regarding the effects of FRDA on all aspects of life. These common viewpoints are illustrated by the following quote:

It's good to have more-- almost a heart. You know. Feel people's suffering. You know, pick up on what is going on in someone's life. (male, age 27 years)

## Discussion

Transitional events identified in this study represent times of change brought about or altered by Friedreich ataxia. Consideration of identified events, coupled with findings from the existing literature, provide insight into the nature and meaning of transitional events, as well as the process of adaptation to these times of change.

### The nature of change

#### An integrated experience

While the distinction between developmental and disease-related transitional events has been made throughout this study, the results may provide clarification of the relationship between these two types of events. Prior research has focused on either disease-related or developmentally-related transitions, but rarely has given simultaneous attention to both types of change. Respondents freely described both disease related and developmental transitions with equal frequency. This distribution of responses draws attention to the widespread effects of FRDA. Participants reported that physical changes correlated with both disease and developmental transitions. Specifically, FRDA creates disease-related transitions that able-bodied individuals do not experience, and also alters most life transitions universally experienced. In many cases, the two types of events were so intertwined, they were indistinguishable to participants.

The separation of disease and developmental events clarifies and characterizes transitional events for research and descriptive purposes. This distinction may also occur in the clinical care of patients. Specifically, care offered to patients typically focuses on the physical aspects of the condition, leaving little opportunity for the individual to explore the totality and interconnected nature of life changes resulting from his/her illness or disability [31].

#### Developmental transitions

Developmental events identified in this study reiterated those described in the literature. Catanzaro [32] reported that chronic illness and disability have significant influences on three major components of one's life: relationships, parenting and career. These components were investigated in individuals with various progressive, neurological conditions. Not only were the areas of change (i.e. relationships and career) comparable to those identified in the present study, but the specific nature of change was also similar. Changes in relationships resulting from incongruence in disease perception, decreased participation in children's activities, and early retirement, were identical in both studies. These similarities may suggest that many developmentally-related events are comparable regardless of the specific progressive, neurological condition.

Not surprisingly, developmentally-related transitional events reported in the present study are similar to transitions identified in healthy persons [32,33]. The significant areas of change identified in this study (relationships and career) have been reported as the primary areas of transition within the general population [33]. However, the nature of the transitional events was quite distinct from those without chronic illness. Developmental transitions identified in the present study population were significant because the effects of FRDA were perceived to alter the "normal" course of life changes.

Overall, the present study lends support to existing literature regarding developmentally-related transitional events in the context of progressive, neurological conditions. Data suggest that progressive, neurological conditions increase the complexity of significant developmental changes. Ultimately, this increased complexity, coupled with a greater magnitude of events brought about by progressive nature of the condition, leaves affected individuals to face a cyclical and perpetual pattern of change. This experience is distinct from that described in the general population where periods of stability are predominant and only occasionally are interspersed with times of transition [6].

#### Disease-related transitions

Unlike developmentally-related transitions, it is difficult to compare FRDA disease-related transitions to the existing literature, as many of these changes are inherently dependent on the clinical manifestations of the condition [9,12]. As a result, research has primarily focused on understanding psychological transitions, or adaptation processes, rather than specific disease-related transitional events.

In the limited studies investigating specific disease-related transitions in chronic illness and disability, diagnosis has been identified as a key event, perhaps because of its near universality within the disease experience [9,12]. Participants in the present study did not report diagnosis as a key transition, but rather focused on their initial recognition of symptom onset. Diagnosis may not have been prominent because most participants were previously aware of their symptoms. Thus, a diagnosis may not always be significant, and its relevance may correspond temporally with the progression of the condition.

Overall, the significant disease-related transitional events, including recognition of symptoms, fear of falling, and changes in mobility status, correspond with the clinical progression of FRDA. A similar relationship between transitional events and clinical progression was also noted in a study of individuals with various forms of muscular dystrophy [12]. The authors of that study characterized the progression as a transition from independence to dependence [12]. In the present population of FRDA participants, a trend toward increasing dependence may be represented early in the progression of the condition. However, movement toward dependence did not continue in a linear fashion, as the use of assistive devices increased one's self reported independence and freedom. Although participants in our study reported an increase in independence, it is unknown how this change affects their quality of life. One study [34] found that motor disability status was linked to decreased quality of life in individuals with adult onset ataxia.

Overall, perceptions reported in the present study regarding disease-related transitional events varied from some previously reported [9,12]. The participants' placed emphasis on recognition of symptoms and increased independence achieved through the use of assistive devices. This focus may suggest that participants have greater concern about the implications of transitions on daily life, than an actual change in status brought about by a diagnosis or assistive device. Additionally, the perceived increase in freedom when using a wheelchair may suggest that independence and dependence are not overall "states of being", nor are they in direct opposition. Rather, these concepts may represent a spectrum of possibilities that are highly situation-dependent [35].

### The meaning of change

Transitional events identified in the present study were characterized by loss, challenges to one's self esteem and alterations in one's self-identity. Not surprisingly, loss and self-identity are recognized as related concepts within the context of chronic illness and disability [36,37]. Chronically ill individuals have been reported to have a heightened sense of concern about the person they are becoming, as well as the loss of self-images from the past [38]. The loss of former images is often not replaced by equally valued new images. This perpetual cycle of loss, termed chronic sorrow, can lead not only to a decline in self-esteem, but eventually to alterations in self-identity [38,39].

Transitional events identified in the present study can be explored in light of the established relationship between loss and self-identity. Loss was primarily brought about by physical changes, discrepancy between perceived and desired self, and stigma. Each form of loss presented specific challenges to one's self esteem, as well as personal identity.

The loss of physical abilities was a focus in many disease-related events. Loss was significant as it led to an inability to perform certain tasks. Since much of an individual's self esteem is derived from one's abilities [11,36], this loss may diminish one's self esteem. Furthermore, changes in one's activities may cause an altered self-identity, as the activities that once contributed to defining self are no longer applicable.

The second form of loss accompanying the reported transitional events resulted from a discrepancy in one's perceived versus desired self. This loss was most poignant within the context of developmentally-related transitional events. Individuals forced to leave a chosen career faced the discrepancy between one's desired self and one's perceived self, who was unable to perform the tasks required for a given field of work. Loss related to developmentally-related events may cause one to be particularly vulnerable to changes in self-perception, as career and relationships have been recognized as the key means by which individuals form their personal identities [33,37].

The final form of loss related to transitional events was that of actual and/or perceived stigma. Participants in the present study reported that many transitional events, specifically changes in mobility status, were accompanied by social stigma. Stigma represented a form of loss, as one was unable to portray him/herself to others in a desired manner. Although clinical manifestations of FRDA were regarded as stigmatizing, individuals often engaged in impression management by developing skills that caused the clinical features not to be noticed by others [37,40]. Stigma related to the use of assistive devices was more prominent for the individual, perhaps because impression management was more difficult to accomplish. Regardless, however, of the stigmatizing stimulus, other's attitudes contribute to self-esteem [36,41,42]. If these attitudes are internalized over a period of time, individuals are likely to experience a change in self-identity from this form of loss.

### Adaptation to change

Overall, transitional life events identified in the present study represent changes brought about or altered by Friedreich ataxia. Adaptation to these changes, and ultimately to the condition is considered critical to the well being of the individual. A prominent literature regarding adaptation in the context of chronic illness and disability has emerged over time and has produced a variety of theoretical frameworks in which to consider this process. Leventhal's self-regulatory model of illness behavior is one such theory in which to consider the present findings regarding transitional events [43].

The overarching premise of Leventhal's model is that individuals respond to change by attempting to re-establish their state of normality [43,44]. In order to accomplish this task, the individual proceeds through three distinct phases. First, one is made aware of the change, and is motivated to assign meaning to the specific problem. In response to this meaning, the individual then identifies and develops potential coping strategies in the attempt to deal with the change. Finally, the individual evaluates his/her strategies and pursues new coping mechanisms if the previous strategies are not considered to be effective.

In keeping with Leventhal's model, an individual seeks to re-establish a state of normalcy in response to change brought about by developmentally and disease-related transitional events. As these events create change, the continuity of one's life is disrupted. In order to re-establish continuity, one begins by assigning meaning to the given event. The individual's ability to interpret experiences within a meaningful framework, also referred to as a sense of coherence, is considered critical to the adaptation process and has been shown to be influenced by the overall stability of one's self-identity [42,45]. It is probable that this phase of the adaptation process is difficult and yet essential for the present study population. Some studies purport that the ability to develop and preserve one's identity is the primary factor in one's eventual adaptation to an event [37,46,47].

Subsequent to this important interpretation phase, individuals begin to utilize a variety of coping strategies to deal with the change. Several previously described strategies were reported by participants in the present study. In the final step of Leventhal's model, the individual begins to appraise and revise his/her coping strategies. This revision component creates a cyclical nature to this model of adaptation, appropriately corresponding to the perpetual nature of change encountered by this study population and highlighting the continual nature of adaptation process throughout the duration of living with FRDA.

#### Implications for genetic counseling

Results from this study have several implications for genetic counseling. First, the findings support previous literature, which indicates that genetic information should be explored with the patient in the broader context of his/her daily life [13,48]. Genetic counselors often provide patients with information about disease-related transitional events, such as diagnosis, prognosis, and genetic status. The significance of this information is dependent upon and highly integrated into many other aspects of life. By recognizing and exploring the far-reaching nature of disease-related transitions, patients may be able to anticipate implications and incorporate and assign meaning to the information in the context of their lives.

Additionally, a genetic counselor can prepare patients for transitions they may encounter in the future, as anticipatory guidance is thought to help facilitate the transition process [27,49]. This preparation may be particularly important due to the complexity of change, as well as the potential for feelings of isolation. Genetic counselors can discuss future transitions with patients, as well as provide referrals for resources and support. Referrals for vocational, and marriage and family counselors, and physical and occupational therapists, may be useful, as indicated by many participants in this study.

Finally, genetic counselors, practicing in settings providing continual, follow-up care to individuals with progressive, neuromuscular conditions, may be able to engage patients in ongoing discussions regarding the development and preservation of self-identity. This process could begin by exploring how patients define their identity (i.e. relationships, career, hobbies, etc), as well as areas in which they enjoy and demonstrate competence. Coupling this information with an exploration of previous or anticipated loss, may allow the genetic counselor and patient to consider meaningful ways in which to replace loss. Ultimately, a demonstration of this process within a counseling session may help the patient in finding ways to develop and restore meaning, control and self-identity in spite of the progressive nature of the condition.

#### Limitations and future research

This study is limited by its selection bias. It is probable that individuals participating in the study are different from those individuals with FRDA who did not participate. Individuals farther along in the progression of the condition, and those having difficulty adjusting to the diagnosis may not have chosen to participate. Furthermore, the populations from which the participants were recruited represent FRDA patients with specific interests. Participants may have been different from non-participants because they are more interested in research or support groups. Results from the study are not generalizable to the overall population of individuals affected with FRDA. The qualitative study design was intended to explore transitional events, and not to test hypotheses or generate generalizable results.

The study is also limited by recall bias. Participants were asked to recall information regarding past transitional events and interactions with health care providers. However, since the primary focus was on the patient's perceptions of transitional events, changes over time are perceived in a broader life span context. Finally, the analysis of the results is also limited in that no comparisons were made between gender, severity, or age of onset: factors that probably would contribute to variation in response.

Wilson et al. [50] found that people with an adult onset of FRDA have a lower quality of life than those who were diagnosed in childhood. It has been speculated that it may be difficult for individuals to adapt to disease transitions after certain aspects of life, such as career and family have been established [50]. Our sample may have recruited more individuals with later onset disease, as we recruited only adults: some of whom participated in the National Ataxia Foundation's conference. Thus, age of our participants may have directly influenced their perception of transitional life events.

Finally, patient perceptions of transitional life events may be altered with new advances in technology. For example, individuals who have taken medications such as idebenone may have a different perception of daily impact of FRDA on their lives. Thus, it is possible that as our understanding and treatment of the disease improves, patients may focus less on disease related transitions described in this study.

Additional research may stem from this study. A quantitative survey assessing the prominent transitional events in progressive neuromuscular conditions would be useful, as it may be able to provide large-scale, generalizable results regarding the relative importance of disease versus developmental transitions, as well as stratify study populations to look for differences in terms of gender, severity and age of onset. Additionally, research regarding the roles, challenges and needs of genetic counselors practicing in settings with continual, follow-up of patients would be beneficial in applying information regarding transitional life events to a clinic setting. Finally, developing and evaluating counseling interventions, which are designed to help facilitate patient adaptation to change, and build self-identity, would also be useful.

## Conclusions

Friedreich ataxia increased the complexity and magnitude of transitional events for study participants. The identified transitional events commonly represented significant loss and presented challenges to self-esteem and identity. Patients with progressive neuromuscular conditions may benefit from genetic counseling strategies which focus on key times of transition and help to establish self-identity throughout the lifespan.

## Competing interests

The authors declare that they have no competing interests.

## Authors' contributions

VBW was involved in the conception of the study, carried out interviews, performed analysis of the data, and drafted the manuscript. JRL participated in the design of the study, helped with recruitment strategies, and assisted with manuscript. JMF also participated in the design of the study, helped with recruitment strategies, and assisted with the manuscript. BBB was involved in the conception and design of the study, performed analysis of the data and edited the manuscript. All authors have read and approve this manuscript.

## Appendix 1 - Summary of transitional events

### Disease-related events

Recognition of symptoms

Self-doubt regarding existence of symptoms

Isolation from support persons when etiology cannot be identified

Fear of falling

Decline in participation in activities

Embarrassment from falling in front of others

Mobility status

Increased freedom with assistive devices

### Developmentally-related events

Relationships

Dating

Difficulty finding individuals to date

Difficulty with discussions regarding the progression of FA

Date's acceptance of FA

Marriage

Important source of support

Difficulty with communication about FA

Adverse effects of FA on the martial relationship

Parenting

Inability to participate in children's activities

Children's need for their parents

The decision not to parent

Life's work

Career choice

Desire verses ability

Job performance

Increased effort and decreased abilities

Early retirement or changing careers

Loss of chosen career
